# Interleukin-7 Unveils Pathogen-Specific T Cells by Enhancing Antigen-Recall Responses

**DOI:** 10.1093/infdis/jiy096

**Published:** 2018-02-28

**Authors:** Nadia Terrazzini, Paola Mantegani, Florian Kern, Claudio Fortis, Anna Mondino, Stefano Caserta

**Affiliations:** 1 School of Pharmacy and Biomolecular Sciences, University of Brighton, United Kingdom; 2 Laboratory of Clinical Immunology, Clinic of Infectious Diseases, San Raffaele Scientific Institute, Milan, Italy; 3 Brighton and Sussex Medical School, The University of Sussex, Falmer, East Sussex, United Kingdom; 4 Division of Immunology, Transplantation and Infectious Diseases, San Raffaele Scientific Institute, Milan, Italy; 5 School of Life Sciences, The University of Hull, United Kingdom

**Keywords:** candida infection, pathogen-specific responses, SEB responses, TB infection

## Abstract

**Background:**

Interleukin (IL)-7 promotes the generation, expansion, and survival of memory T cells. Previous mouse and human studies showed that IL-7 can support immune cell reconstitution in lymphopenic conditions, expand tumor-reactive T cells for adoptive immunotherapy, and enhance effector cytokine expression by autoreactive T cells. Whether pathogen-reactive T cells also benefit from IL-7 exposure remains unknown.

**Methods:**

In this study, we investigated this issue in cultures of peripheral blood mononuclear cells (PBMCs) derived from patients infected with various endemic pathogens. After short-term exposure to IL-7, we measured PBMC responses to antigens derived from pathogens, such as *Mycobacterium tuberculosis*, *Candida albicans*, and cytomegalovirus, and to the superantigen *Staphylococcus aureus* enterotoxin B.

**Results:**

We found that IL-7 favored the expansion and, in some instances, the uncovering of pathogen-reactive CD4 T cells, by promoting pathogen-specific interferon-γ, IL-2, and tumor necrosis factor recall responses.

**Conclusions:**

Our findings indicate that IL-7 unveils and supports reactivation of pathogen-specific T cells with possible diagnostic, prognostic, and therapeutic significance of clinical value, especially in conditions of pathogen persistence and chronic infection.

## Teaser

IL-7 supports host immunity in lymphopenic and immunosuppressed patients and expands tumor-reactive T cells for adoptive immunotherapy. Here, we report that IL-7 also enables the accumulation of clinically relevant pathogen-reactive T cells from TB-, CMV-, and *Candida albicans*-infected individuals.

Interleukin (IL)-7 [1] is a pleiotropic cytokine [2] regulating lymphopoiesis and T-cell homeostasis [3, 4]. It binds to a heterodimeric receptor formed by an α-chain (CD127 [3]), which is private to the IL-7 receptor (IL-7R) and the common-γ-chain cytokine-receptor (CD132 [5, 6]). In humans, mutations negatively affecting the levels of CD127 have been correlated with severe immunodeficiency [7]. Conversely, increased serum levels of IL-7 and/or dysregulated activation of CD127 are reported in patients with autoimmune conditions, including multiple sclerosis [8, 9], rheumatoid arthritis [10], type-I diabetes [11], inflammatory bowel disease [12], and psoriasis [13].

Upon antigen (Ag) encounter, IL-7 sustains the generation of memory T lymphocytes in vitro [14] and in vivo [14–16] and favors the transition of effector to central memory cells [17, 18] while driving their proliferation [19]. Via the JAK3/STAT5 pathway and the upregulation of antiapoptotic factors (eg, Bcl-2) [14, 20], IL-7 promotes the long-term survival of naive and memory-phenotype cells. Recombinant IL-7 elicits a marked increase of central memory (T_CM_) and effector memory T cells (T_EM_) when administered to aged nonhuman primates [21] and lymphopenic patients and macaques infected with human immunodeficiency virus (HIV) or simian immunodeficiency virus, respectively [22–24]. Similar effects are reported in patients undergoing stem cell transplantation [25, 26], where IL-7 levels correlate with the generation of stem-cell memory T cells [26]. In cancer patients, IL-7 preferentially increases naive but not regulatory T-cell numbers [27], maintaining T-cell receptor (TCR) repertoire diversity [28]. Hence, the immunotherapeutic use of IL-7 is increasingly proposed to favor immune-cell reconstitution and function after lympho-depleting chemotherapy or in the elderly. In the setting of adoptive T-cell therapy (ACT), IL-7 has been used in primary cultures to engineer and expand tumor-reactive T cells [29, 30]. We previously found that IL-7 selectively expands tumor-reactive CD4 T cells capable of promoting tumor protection in ACT [29]. Whether similar results could be extendible to pathogen-specific T cells remains unknown. Therefore, we sought to investigate the expansion of pathogen-reactive CD4 T cells in individuals affected by recurrent or persistent/chronic bacterial (*Mycobacterium tuberculosis* [MTB] and *Staphylococcus aureus*), fungal (*Candida albicans* [Ca]), or viral (cytomegalovirus [CMV]) infections. We report that, in all cases, IL-7 enriched pathogen-specific CD4 T cells, enabling their detection and sensitizing them to Ag-specific recall responses. Furthermore, IL-7 rescued chronically activated pathogen-specific effectors enhancing their Ag-recall responses. We believe that these data open new avenues for diagnostic, prognostic, and therapeutic applications.

## MATERIALS AND METHODS

### Classification of Tuberculosis Patients

Human immunodeficiency virus-seronegative patients with active tuberculosis ([TB] clinic and culture confirmed) were recruited at the Clinic of Infectious Diseases, San Raffaele Hospital (Milan, Italy). They underwent tuberculin skin testing (TST) administered by the Mantoux method with 0.1 mL (5 tuberculin units) of Biocinetest-PPD tuberculin (Chiron Italia, Milan, Italy). The size of induration was evaluated after 48–72 hours (an induration ≥10 mm was classified as positive). Peripheral blood was drawn before starting any therapy and following written informed consent. Healthy controls were selected among HIV-seronegative individuals with no history of TB exposure, no infection, and with negative reaction to the TST. Healthy controls were tested for the presence of Ca-Ag responses.

### Ethics Statement

Written informed consent or consultee approval to enroll was secured for all study participants (patients and healthy donors). This study was approved by the Ethical Committee of the San Raffaele Scientific Institute, the UK National Research Ethics Service (reference: 13/LO/1270), and the Brighton and Sussex Medical School (BSMS) Research Governance and Ethics Committee (reference: 13/182/LLE) and carried out in accordance with the approved guidelines. All data were anonymized.

### Cytomegalovirus Serology

Healthy donors were screened for the presence of CMV-specific antibody in serum. Cytomegalovirus immunoglobulin (Ig)G serology (Architect CMV IgG; Abbot, Maidenhead, UK) was performed at the Brighton and Sussex University Hospital Trust virology laboratory. Cytomegalovirus-seropositive and -seronegative individuals are referred to as CMV^+^ and CMV^−^, respectively.

### Human Samples and T-Cell Cultures

Patients and healthy donors used for MTB- and *Candida*-specific responses were part of a previously published cohort [31]. Donors (male/female, 10:9) used for CMV and *Staphylococcus aureus* enterotoxin B (SEB)-specific responses were 68 ± 17 years old. Peripheral blood mononuclear cells (PBMCs) were isolated by blood centrifugation over Ficoll-Hypaque (Sigma/Merck, Darmstadt, Germany) density gradient. Cultures were derived either from freshly isolated (CMV/SEB responses) or cryopreserved (90% fetal calf serum [FCS] and 10% dimethyl sulfoxide; MTB/Ca-Ag responses) PBMCs. Equal numbers of viable (0.1% Trypan blue-negative) cells were resuspended in complete media (Roswell Park Memorial Institute medium containing penicillin, streptomycin, glutamine, and 10% FCS [all from Thermo Fisher Scientific] or autologous serum) with or without human recombinant IL-7 (50 ng/mL, unless specified otherwise in individual figures; R&D Systems/Biotechne, Minneapolis, MN) for 7 days. Where indicated, cells were stained with the fluorescent dye 5-(and-6)-carboxyfluorescein diacetate succinimidyl ester ([CFSE] Thermo Fisher Scientific; 1 µM), in accordance with the manufacturer’s instructions, using autologous serum or FCS to quench the labeling. Where indicated, cells were first cultured in complete medium with or without bacterial SEB (1 μg/mL, Sigma/Merck). After 5 days, cells were harvested, washed and counted. Equal number of viable cells were finally seeded in culture with or without IL-7, for additional 7 days (day 12). Cyclosporine A ([CSA] 0.5 µg/mL; Calbiochem/Merck) or anti-lymphocyte function-associated antigen (LFA)-1 blocking antibody (5 µg/mL; a gift from Professor Ruggero Pardi, University Vita-Salute San Raffaele, Milan) were added to the cultures.

### Mycobaterium tuberculosis Peptides and Candida albicans Antigen-Specific Enzyme-Linked Immunospot Assay

The enzyme-linked immunospot (ELISPOT) assay for interferon (IFN)γ detection was performed as described previously [31]. In brief, equal numbers of viable cells (5 × 10^4^ cells/well) were seeded in duplicate in 96-well plates (MAIPS4510; Millipore/Merck), precoated with anti-IFNγ capture monoclonal antibody ([mAb] B-B1; Diaclone, Besançon, France), together with autologous irradiated PBMCs (5 × 10^4^ cells/well), and *Mycobacterium tuberculosis* peptides ([MTPs] a pool of 6 synthetic peptides, 2 µg/mL per peptide [Primm, Milan, Italy]) or Ca-Ag (25 µg/mL [Bio-Rad, Hercules, CA]) for 18 hours at 37°C, in 5% CO_2_ atmosphere. Biotinylated anti-IFNγ detection mAb (B-G1; Diaclone) was added (4 hours), followed by the streptavidin-alkaline phosphatase conjugate (1 hour) (Amersham Pharmacia Biotech Europe GmbH, Freiburg, Germany). After a washing step, the nitroblue tetrazolium-5-bromo-4-chloro-3-indolylphosphate (Sigma) chromogenic substrate was added. Individual spot-forming cells were counted using an automated image analysis system ELISPOT reader (AID-GmbH, Strassberg, Germany). *Mycobacterium tuberculosis* peptides (20 amino acids) were derived from the ESAT-6 and CFP-10 secretory proteins of MTB, purified (>70%), and previously validated [31]. Peripheral blood mononuclear cells in medium alone or stimulated with phytohemagglutinin ([PHA-P] Sigma; 5 µg/mL) were used to assess assay background and functionality.

### Flow Cytometry Analysis and Intracellular Cytokine Staining

Cells were harvested, washed with staining buffer (phosphate-buffered saline supplemented with 0.5% FCS and 0.02% NaN_3_), and incubated with mAbs directed against surface Ags for 15 minutes at room temperature. The following fluorescence-conjugated mAbs were used: anti-CD3-v500, anti-CD8-allophyocyanine-H7, anti-CD27-phycoerythrine (PE), IL-2-fluorescein isothiocyanate, TNFα Alexa700 (all from BD Biosciences, Franklin Lakes, NJ); anti-CD4-peridinin chlorophyll, anti-IFNγ PE-Cy7 (Cyanine-7), anti-CD154 Pacific-Blue (BioLegend, Cambridge, UK); anti-CD45RA ECD (Beckman Coulter, UK); and Yellow live-dead stain (Thermo Fisher Scientific). Thereafter, cells were washed in staining buffer before acquisition.

Intracellular cytokine staining was used to measure MTP-specific cytokine release at single-cell level. Equal numbers of CFSE-labeled cytokine-cultured cells (0.6 × 10^6^) were stimulated (6 hours) with unpulsed (nil) or MTP-pulsed (4 µg/mL) autologous irradiated (5000 rad) PBMCs (3 × 10^6^ cells), in the presence of human anti-CD28-stimulating mAb (2 µg/mL; BD Biosciences). In the last 5 hours of stimulation, Brefeldin A (10 µg/mL; Sigma) was added. To measure CMV-/SEB-specific cytokine release, equal numbers of cells were stimulated with CMV lysate (2 µg/mL; Advanced Biotechnologies, Eldersburg, MD) or SEB (1 µg/mL) for 2 hours followed by 14 hours in the presence of Brefeldin A. Thereafter, cells were washed, surface-stained as described above, fixed, permeabilized, and stained with anti-CD4, anti-IL-2, anti-IFNγ, and/or anti-TNFα mAbs. Events were acquired on a BD FACSCalibur or LSRII, and data were analyzed using the FlowJo-v9.x software (TreeStar Inc., Ashland, OR).

### Statistical Analysis

GraphPad Prism 7.03 was used for statistical analyses. The D’Agostino-Pearson and Shapiro-Wilk tests were used to determine normality of data distribution. For normally distributed data that passed both tests, means and standard deviation are shown, and paired *t* tests are used to compare 2 groups. For non-normally distributed data, non-parametric paired tests (Wilcoxon test) were used to compare 2 groups. For multiple-group comparisons, 2-way ANOVA with Sidak’s or Tukey’s multiple comparison corrections were used, as indicated in the figures. Levels of significance are as follows: *, *P* ≤ .05; **, *P* ≤ .005; and ***, *P* ≤ .0005, unless specified differently in individual figures.

## RESULTS

### Interleukin-7 Enhances Recall Responses of Mycobaterium tuberculosis-Specific CD4^+^ T Cells

**Figure 1.**
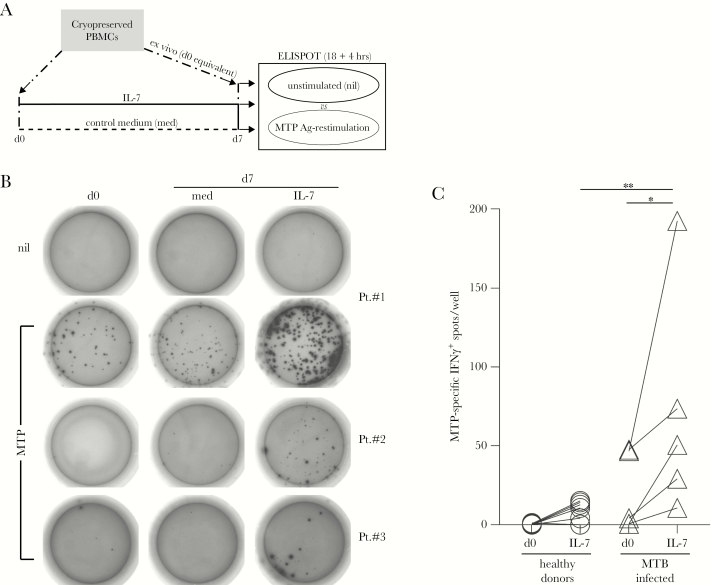
Interleukin (IL)-7 enhances *Mycobacterium tuberculosis* (MTB)-specific T-cell responses. (A) Cryopreserved peripheral blood mononuclear cells (PBMCs) from tuberculosis (TB) patients were analyzed for interferon (IFN)γ release by MTB-specific peptide (MTP) enzyme-linked immunospot (ELISPOT) assay at the time of thawing (day [d]0) and after a 7-day culture in the absence (control medium [med], dashed line) or presence of human recombinant IL-7 (solid line), in parallel to unstimulated controls (nil). For each patient, 2 duplicate vials were sequentially thawed, staggered in time (dash dotted lines): the first vial was used to set up the cultures at d0 (left dashdotted arrow); the second vial was thawed 7 days later (right dash dotted arrow) to provide for the following: (1) ex vivo control cells to be analyzed in parallel to cultured cells and (2) autologous feeder cells for the restimulation assays (refer to Materials and Methods for details). (B) Background IFNγ release was measured for any condition in antigen (Ag)-unpulsed control wells (nil), as representatively depicted for patient (Pt.)#1 (top row). The IFNγ-spots after restimulation with MTP promiscuous peptides were detected in 3 TB patients (Pt. #1, #2, and #3) after thawing (d0) and/or a 7-day culture (d7) in IL-7 or med. (C) The effect of the IL-7 treatment (d0 vs d7, IL-7) on MTP-specific IFNγ-spot accumulation in PBMCs derived from healthy donors (open circles, n = 8) and MTB-infected patients (open triangles, n = 5) was analyzed by the ELISPOT assay in multiple individuals. Statistical significance was determined using a 2-way analysis of variance with Sidak’s multiple comparison correction to evaluate (1) the effect of IL-7 treatment in the 2 different groups over time and (2) the diagnostic power of the discrimination of the 2 groups before and after IL-7 treatment, as indicated: *, *P* ≤ .05; **, *P* ≤ .005.

To investigate putative effects of IL-7 on pathogen-specific T cells, we first analyzed chronically infected TB patients. These were chosen based on clinical history and manifestation of acute MTB infection (clinic and culture confirmed), positive reaction to the TST, and ability of PBMCs to respond to stimulation with major histocompatibility complex-II-restricted MTB-specific promiscuous peptides (MTPs) in IFNγ ELISPOT assays [31]. Patients with detectable (patient [Pt]#1 and Pt#2; Figure 1) or undetectable (Pt#3; Figure 1) MTB-specific IFNγ-producing T cells were analyzed. Cryopreserved cells were tested in MTP-recall assays immediately after thawing (day [d]0; Figure 1A and B) or at the end of a culture (d7) in IL-7 compared with complete media (med). Not all patients with detectable MTP-specific T-cell responses on fresh PBMCs [31] had a detectable MTP response after thawing, as seen in the case of Pt#2, compared with Pt#1. Nonetheless, we found that IL-7 selectively enriched cultures for MTP-specific IFNγ^*+*^ T cells by 4- to 10-fold in all cases (Figure 1B). Although MTP-specific T cells could also expand in control cultures (as seen for Pt#1, med), this was best explained by the increase of total CD3^+^CD4^+^ T cells in some patients (data not shown). It is important to note that the frequency of MTP-specific cells increased in IL-7 cultures more than the levels found in control cultures, even if the percentage of total CD3^+^CD4^+^ T cells remained similar. Of note, IL-7 also allowed us to detect MTP-specific T cells in samples derived from immunosuppressed patients (ie, anergic; Pt#3). Sensitization with IL-7 significantly increased absolute numbers of MTP-specific T cells in all the TB patients analyzed (n = 5; Figure 1C) compared with non-Bacillus Calmette-Guérin-vaccinated healthy donors (non-MTB-infected controls, n = 8).

To independently confirm the presence of MTP-specific T cells, we additionally performed ICS after MTP stimulation. *Mycobacterium tuberculosis* peptide-pulsed irradiated autologous PBMCs were used as Ag-presenting cells (APCs). As with ELISPOT assays, in ICS we found that the frequency of MTP-specific, IL-2^+^IFNγ^+^ CD4 T cells increased in IL-7 cultures, compared with freshly thawed PMBCs (d0) and control cultures (med; Supplementary Figure 1). Thus, MTB-specific T-cell recall responses are enhanced after sensitization with IL-7.

### Interleukin-7 Supports Fungal and Viral-Specific CD4 T-Cell Accumulation in Peripheral Blood Mononuclear Cells From Infected Individuals

**Figure 2.**
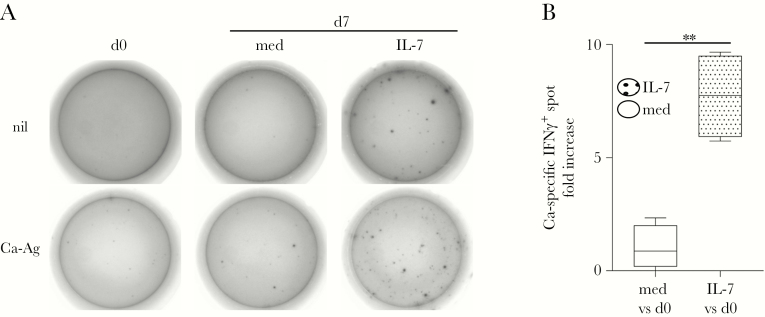
Interleukin (IL)-7 supports the expansion and effector function of *Candida albicans* (Ca)-specific T cells. (A) After thawing, peripheral blood mononuclear cells from a donor with recurrent Ca infection were analyzed for the release of the effector cytokine, interferon (IFN)γ, using a Ca-antigen (Ag)-specific enzyme-linked immunospot assay at the time of thawing (day [d]0) and after a 7-day culture in the absence (d7, control medium [med]) or presence of human recombinant IL-7 (d7, IL-7). Background IFNγ release was measured in Ag-unpulsed control wells (nil, top wells) in the presence of irradiated autologous feeder cells. (B) The range and average fold increase of Ca-Ag-specific IFNγ spots in control medium (med vs d0, white bar) and IL-7 (IL-7 vs d0, dotted bar) cultures over the levels found after thawing (d0) is shown. Statistically significant accumulation of Ca-Ag-specific IFNγ-spots was evaluated in multiple donors (n = 4) using a paired *t* test. *P ≤ .05; **, *P* ≤ .005; ***, *P* ≤ .0005.

We then asked whether exposure to IL-7 would also drive the accumulation of CD4 T cells specific for fungal Ags derived from endemic pathogens, such as Ca. Freshly thawed PBMCs derived from individuals with recurrent Ca infections were immediately analyzed for IFNγ expression in Ca-Ag-recall ELISPOT, in the presence of Ag-pulsed irradiated autologous PBMCs as APCs (d0; Figure 2), or seeded in culture with or without IL-7 (d7 and med, respectively; Figure 2), and analyzed after 7 days. As in the case of TB-specific T cells, Ca-Ag-specific IFNγ spots were also remarkably increased in IL-7-driven cultures, compared with controls (d0 and med; Figure 2A). In multiple individuals (n = 4), Ca-Ag-specific effectors were significantly increased by ~10-fold over the levels found at d0 after IL-7 exposure compared with control medium (Figure 2B).

**Figure 3.**
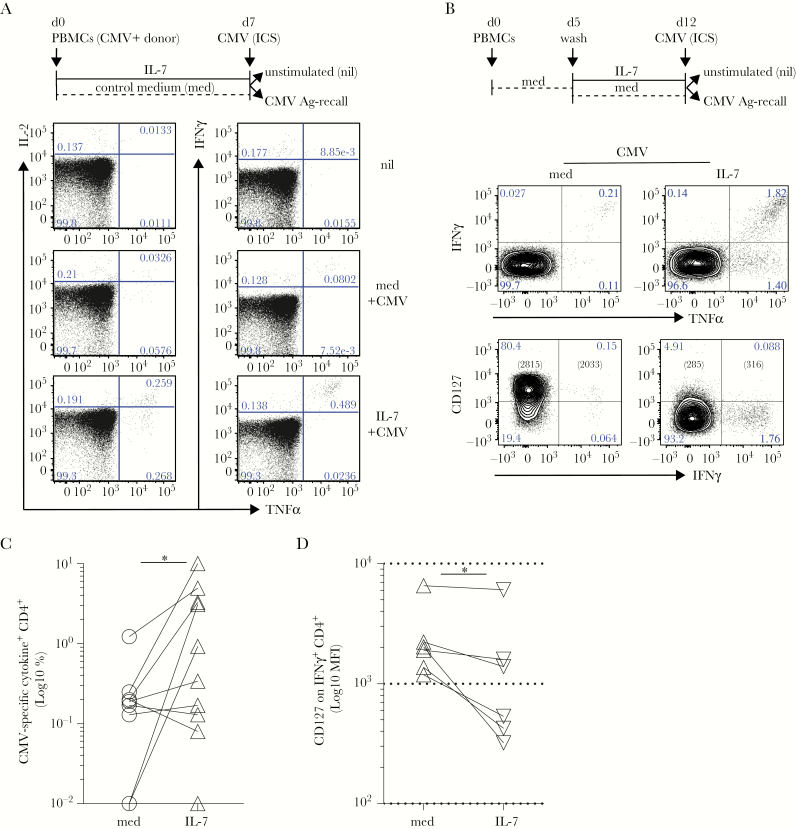
Interleukin (IL)-7 supports the accumulation of antiviral cytomegalovirus (CMV)-specific T cells. (A) Freshly derived peripheral blood mononuclear cells (PBMCs) from CMV^+^ donors were analyzed for inflammatory cytokine release after CMV-lysate intracellular cytokine staining (ICS) assay after a 7-day culture in the absence (d7, control medium [med], dashed black line) or presence of human recombinant IL-7 (d7, IL-7, black line). Left and right dot plots show the levels of IL-2 and tumor necrosis factor (TNF)α or interferon (IFN)γ and TNFα in gated CD4 T cells, respectively. Background levels of cytokine secretion were typically measured in unstimulated controls (nil). (B) Freshly isolated PBMCs from CMV^+^ donors were rested for 5 days in plain medium (dashed black line) before a 7-day culture in the absence (d12, med, dashed black line) or presence of human recombinant IL-7 (d12, IL-7, black line). At day 12 (d12), CD4^+^ T cells were analyzed for IFNγ and TNFα release (top row) alongside expression of CD127 (bottom row), after CMV-lysate ICS assay. Cytomegalovirus-specific IFNγ^+^ CD4^+^ T cells show high CD127 expression in the resting cultures, whereas they downregulated CD127 expression upon exposure to IL-7. Background levels of cytokine secretion were typically measured in ICS unstimulated (nil) controls. A representative of >10 independent experiments is shown. (C) After subtraction of individual background levels of IFNγ^+^ and TNFα^+^ and IFNγ^+^TNFα^+^ CD4 T cells detected in unstimulated controls (nil), the percentage of CMV-specific cytokine^+^ (IFNγ^+^ and TNFα^+^) CD4 T cells was evaluated in 10 independent CMV^+^ donors in IL-7 (IL-7, open triangles) compared with control medium ([med], open circles) cultures. The graph shows a statistically significant (paired Wilcoxon test, *P = .03) increase of the frequency (Log_10_) of CMV-specific CD4 T cells after IL-7 culture. (D) CD127 expression (Log_10_ mean fluorescence intensity [MFI]) is significantly downregulated in CMV-specific IFNγ^+^CD4^+^ T cells exposed to IL-7 (IL-7) compared with control medium (med) at d12 in 6 biologically independent replicates (paired Wilcoxon test, *P = .03).

Because CMV-specific CD4 T cells have been recently shown to significantly expand in elderly individuals chronically infected with the endemic CMV [32, 33], we investigated CMV-specific CD4 T-cell responsiveness to IL-7 in cultures derived from CMV-seropositive (CMV^+^) patients. CMV-seronegative (CMV^−^) individuals were used as controls. Freshly derived PBMCs were cultured in the presence of IL-7 or control medium (med) for 7 days, and then CMV-recall responses were tested in ICS, using total CMV lysate as a source of viral Ags (Figure 3A, top). Again, cells cultured in IL-7 tended to show higher CMV-recall responses compared with controls (Figure 3A). Of note, these CMV-specific T cells were mostly polyfunctional, TNFα^+^IFNγ^+^-producing cells (Figure 3A) and remained undetectable in cultures derived from CMV^−^ individuals (Supplementary Figure 2A). Because we found that T cells express varying amounts of CD127 and upregulate it in control medium (Supplementary Figure 2B), as expected [34], we first rested freshly thawed PBMCs for 5 days, and then we subjected them to IL-7-driven cultures (Figure 3B, top). Under these conditions, we found that IL-7 more potently and reproducibly enabled the accumulation of CMV-specific T-cell responses from CMV^+^ individuals (n = 10; Figure 3C). The IFNγ^+^ cells expressed low levels of CD127, indicative of IL-7-driven receptor downmodulation [34], upon cytokine-driven activation (Figure 3D) while maintaining high levels of LFA-1 (data not shown) and polyfunctional cytokine expression (Figure 3B). Thus, in addition to bacterial responses, fungal and viral-specific responses are also enhanced by IL-7 signals.

### Interleukin-7 Promotes Responsiveness of *Staphylococcus aureus*- Specific T Cells

**Figure 4.**
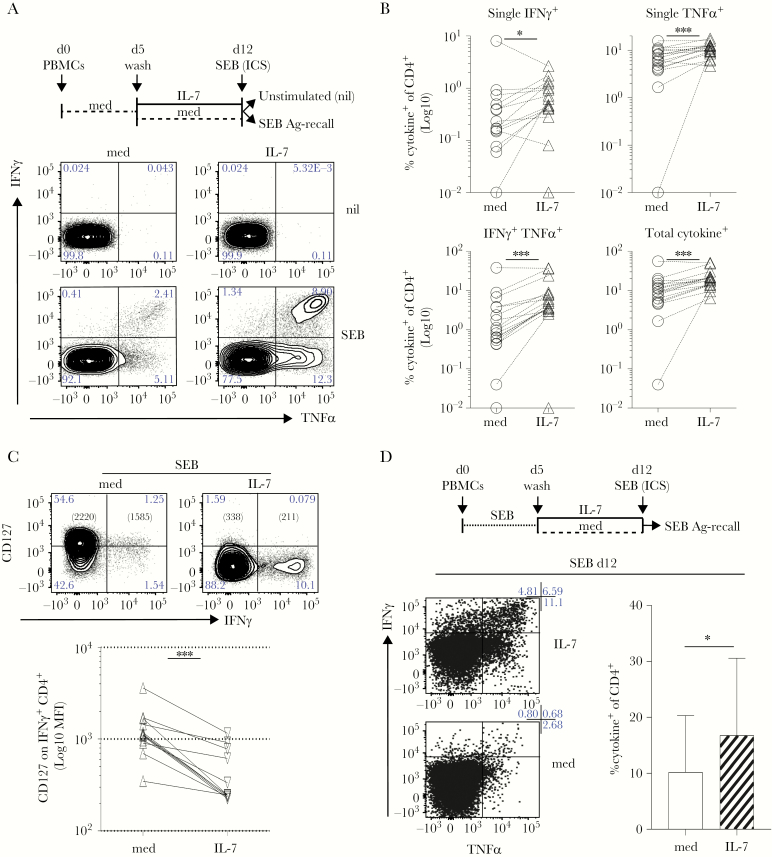
Interleukin (IL)-7 supports superantigen-specific responses. (A) Freshly isolated peripheral blood mononuclear cells (PBMCs) were rested for 5 days in plain medium (dashed black line) then incubated for 1 week in IL-7 or control medium ([med] respectively IL-7, black line and med, dashed black line). At day (d)12, cells were stimulated with Staphylococcus aureus enterotoxin B (SEB) followed by an intracellular cytokine staining (ICS) assay to test antigen (Ag)-specific tumor necrosis factor (TNF)α and interferon (IFN)γ release, compared with unstimulated controls (nil). Dot plots show that the frequency of SEB-specific CD4^+^ T cells producing TNFα and/or IFNγ increased upon exposure to IL-7 in 1 representative donor. (B) After subtraction of individual background levels of IFNγ^+^ and TNFα^+^ and IFNγ^+^TNFα^+^ CD4 T cells detected in unstimulated controls (nil), the percentage of SEB-specific cytokine^+^ (IFNγ^+^, TNFα^+^, IFNγ^+^TNFα^+^ and total) CD4 T cells was evaluated in 17 independent donors in IL-7 (IL-7, open triangles) compared with control medium (med, open circles) cultures. The graphs show a statistically significant increase of the frequency (Log_10_) of SEB-specific cytokine^+^ CD4 T cells after IL-7 culture. All tests are paired Wilcoxon tests with the exception of the single TNFα^+^ analysis (paired *t* test): *, *P* ≤ .05; **, *P* ≤ .005; ***, *P* ≤ .0005. (C) At d12, cells cultured as in (A) were tested for CD127 expression in parallel to cytokine release in SEB-specific ICS assay. CD127 expression (Log_10_ mean fluorescence intensity [MFI]) is significantly decreased in SEB-specific IFNγ^+^ CD4^+^ T cells exposed to IL-7 (IL-7, downward triangles) compared with control medium (med, upward triangles) in 12 independent biological replicates (paired Wilcoxon test; ***, *P* = .0005). (D) Freshly isolated PBMCs (d0) were stimulated with the SEB superantigen (dotted black line) for 5 days (d0–5). At d5, cells were washed and incubated for 1 week in IL-7 (IL-7, black line; d5–12) or control medium (med, dashed black line; d5–12). At d12, cells were stimulated with SEB (overnight) to test Ag-specific TNFα and IFNγ release, by ICS. Dot plots show that the frequency of SEB-specific CD4^+^ T cells producing TNFα and/or IFNγ increased upon exposure to IL-7 (IL-7 d5–12, top row). The graph on the right shows data from the same cultures derived from 6 independent biological replicates. Statistically significant accumulation of SEB-specific, cytokine^+^ CD4^+^ T cells was evaluated using a Wilcoxon matched-pairs signed-ranked test (*P* = .03).

Bacterial superantigens, such as SEB, can lead to T-cell anergy [35] or suppression [36]. We asked whether culturing the cells in IL-7 might also help SEB-specific T-cell responses (Figure 4). The PBMCs from healthy donors were rested 5 days in control medium and then left untreated or cultured in IL-7 for 7 days. The SEB-recall responses were then tested in ICS (Figure 4A, top). We found that although SEB-specific T cells could be detected in control PBMC cultures (med), IFNγ^+^ or TNFα^+^ and TNFα^+^IFNγ^+^ cells were all enriched for in IL-7-driven cultures from many individuals, significantly (Figure 4A and B, n = 17). As seen for CMV-specific T cells (Figure 3D), cytokine^+^ SEB-specific cells expressed lower levels of CD127 (Figure 4C) while maintaining high levels of LFA-1 (data not shown).

We also tested whether IL-7 is capable of supporting SEB-responsiveness even after Ag-driven expansion, in vitro (Figure 4D). We found that IL-7 significantly enhanced responses after SEB-restimulation (Figure 4D). Thus, culturing T cells in IL-7 either before or after Ag recall allows for a higher frequency of Ag-specific cells to be identified.

### Interleukin-7 Drives Cyclosporine A-Sensitive Central Memory T-Cell Division

**Figure 5.**
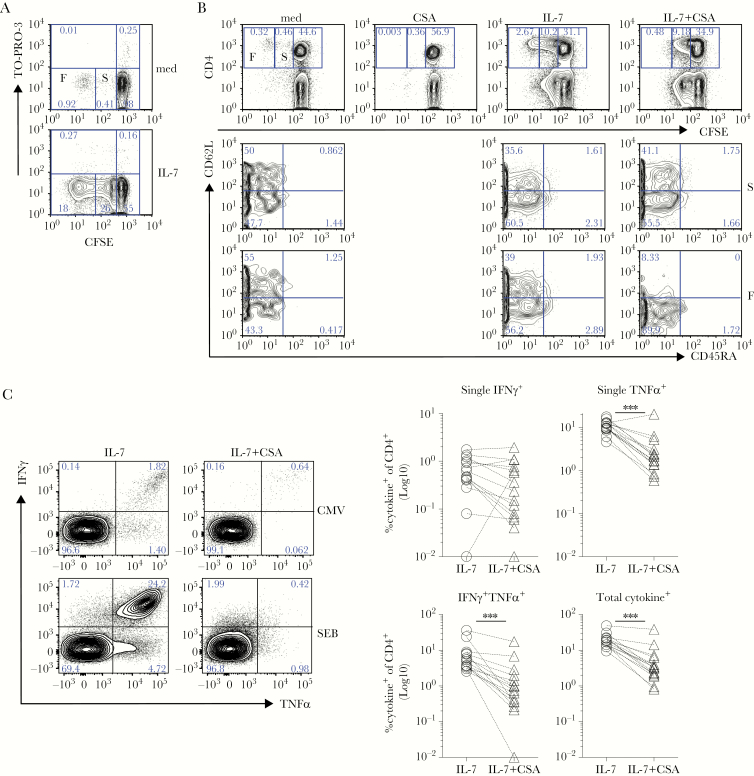
Sensitization by interleukin (IL)-7 promotes pathogen-specific CD4^+^ T cell proliferation in a cyclosporine A (CSA)-sensitive manner. 5-(and-6)-carboxyfluorescein diacetate succinimilyl ester (CFSE)-labeled peripheral blood mononuclear cells (PBMCs) (A–C) from healthy donors (A and B) were cultured for 7 days in the absence (med) or presence of human recombinant IL-7 (IL-7). (A) Proliferation of viable CD4 T cells in high-density cultures (5 × 10^6^ cells/mL) was determined by flow cytometry after staining with anti-CD4 monoclonal antibody (mAb) and TO-PRO-3 (an intercalant agent of deoxyribonucleic acid entering necrotic and apoptotic cells). Contour plots depict the relative CFSE content within the same number of viable, TO-PRO-3^−^ CD4^+^ T cells. (B) Cells cultured (4 × 10^6^ cells/mL) in the presence of IL-7 for 7 days in the absence or in the presence of CSA. At d7, cells were stained with anti-CD4, anti-CD45RA, and anti-CD62L mAb, and the relative CFSE content was analyzed by flow cytometry in total CD4 T cells (top row). Fast- (F), slow- (S), and nondividing cells were determined based on CSA inhibition, as indicated. The percentage of naive (CD45RA^+^CD62L^hi^), central memory (CD45RA^−^CD62L^hi^), and effector memory (CD45RA^−^CD62L^lo^) cells were then investigated in slow- compared with fast-proliferating CD4 T cells. (C) Freshly isolated PBMCs were rested for 5 days then incubated for 1 week in IL-7 or IL-7 plus CSA (IL-7+CSA). At d12, cells were stimulated with *Staphylococcus aureus* enterotoxin B (SEB) during an intracellular cytokine staining (ICS) assay to test antigen-specific tumor necrosis factor (TNF)α and interferon (IFN)-γ release, compared with unstimulated controls (refer to Figures 3 and 4). Left dot plots show that the IL-7-driven accumulation of (top) cytomegalovirus (CMV)- and (bottom) SEB-specific CD4^+^ T cells producing TNFα and/or IFNγ is reversed in the presence of CSA, by d12. After subtraction of individual background levels of IFNγ^+^, TNFα^+^, and IFNγ^+^TNFα^+^ CD4 T cells detected in unstimulated controls (nil), the percentage of SEB-specific cytokine^+^ (IFNγ^+^, TNFα^+^, IFNγ^+^TNFα^+^, and total) CD4 T cells was evaluated in 15 independent biological replicates in IL-7 (open circles) compared with IL-7+CSA cultures (open triangles). The right graphs show a statistically significant decrease in the frequency (Log_10_) of SEB-specific cytokine^+^ (except for IFNγ^+^) CD4 T cells in the presence of CSA at d12. All tests are paired Wilcoxon tests; *, *P* ≤ .05; **, *P* ≤ .005; ***, *P* ≤ .0005.

In a previous mouse study, we found that IL-7 favored the in vitro expansion of tumor-Ag-experienced T cells, by promoting their proliferation [29]. Thus, we asked whether IL-7-driven accumulation of human, pathogen-specific T cells also requires cell division. To this aim, we set up CFSE-labeled PBMC cultures (Figure 5A and B). We found that although a small fraction of CD4 T cells derived from healthy donors (~1%–10%) underwent several rounds of “spontaneous” (ie, in the absence of any introduced stimulation) cell division in complete media (Figure 5A, top panel), dividing cells were dramatically increased upon IL-7 addition (Figure 5A, bottom panel). These cells expressed high levels of Bcl-2 (Supplementary Figure 3), in agreement with the prosurvival role of the cytokine [20]. More importantly, IL-7 favored the expansion of 2 cell populations, distinguishable for their proliferation potential (ie, either fast or slow; F and S, respectively, in the figures) and with distinct mechanistic requirements. Indeed, although both populations were best detected in high-density cell cultures (Supplementary Figure 4), they proved differentially sensitive to CSA. Fast- but not slow-dividing cells were sensitive to CSA inhibition (Figure 5B). In addition, fast-dividing cells were sensitive to anti-LFA-1 mAb blockade (Supplementary Figure 5), suggesting that cell-to-cell contacts are also required for their proliferation. Of note, IL-7 elicited similar effects in autologous sera and FCS (Supplementary Figure 4). We reckoned that, although slow-dividing cells represent cells undergoing homeostatic expansion, known to occur via CSA-insensitive mechanisms [19] (Figure 5B, right panel in top row), fast-dividing cells may represent a distinct population of Ag-experienced memory cells proliferating via a CSA-sensitive mechanism (Figure 5B, middle-right panel in top row). Flow cytometry analysis indicated that although both T_CM_ (CD45RA^−^, CD62L^+^) and T_EM_ (CD45RA^−^, CD62L^−^) cells accumulated in response to IL-7 (Figure 5B, middle and bottom panel), only fast-dividing T_CM_ (CD45RA^−^, CD62L^+^) cells appeared sensitive to CSA (Figure 5B, bottom panel). It is interesting to note that the IL-7-driven accumulation of CMV (Figure 5C, top), SEB (n = 15; Figure 5C, bottom and right), and MTB-specific (Supplementary Figure 6) polyfunctional memory T cells was also completely dependent upon CSA-sensitive signaling. Taken together, our data indicate that IL-7-driven cultures might recapitulate the events accounting for the maintenance of Ag-experienced memory T-cell subsets in vivo [15] and improve their identification and selection in vitro.

## DISCUSSION

In this study, we demonstrate that IL-7 supports the in vitro expansion of human pathogen-specific T cells, favoring and, in some instances, enabling their enumeration and characterization. Interleukin-7-sensitive pathogen-specific effectors included cells specific for endemically diffused pathogens (eg, *S aureus*, *C albicans*, CMV, and MTB, in parts of the world), infecting humans recurrently during the lifetime, persisting (MTB) and/or reactivating in the body (CMV). Thus, our data will help the study and isolation of such pathogen-specific T cells and others relevant to various clinical settings, including harmful infections, especially in the case of immune-suppressed (HIV, transplantation, aged etc) or chronically infected individuals, in which pathogen-specific T cells might be low in frequency and/or hyporesponsive.

Previous studies showed that IL-7 can support immune-cell reconstitution in lymphopenic conditions [22–25], restore sepsis-induced lymphocyte dysfunctions ([37, 38]), enhance effector function of autoreactive T cells [8–13], and expand tumor-reactive T cells [29, 30]. We then asked whether pathogen-reactive T cells also benefited from IL-7 exposure. Our results show that IL-7 promotes the selective expansion of a fraction of memory CD4^+^ T cells containing pathogen-specific cells, best observed in high cell-density cultures. Although IL-7 significantly enhanced overall cell recovery (by ~1.5-fold), cytokine-producing CD4 T cells were not enriched at a polyclonal level (as detected by 12-*O*-tetradecanoyl-phorbol-13-acetate and ionomycin stimulation; Supplementary Figure 7) unlike that seen for pathogen-specific cells. These cells showed a fast rate of proliferation, sensitive to CSA and LFA-1 inhibition, both in autologous sera and FCS. Thus, neither homeostatic cell division [19, 39–42], known to be CSA insensitive [19, 42], nor food-related, bovine Ags seem to account for the accumulation of fast-dividing cells. However, our results suggest the existence of a cell-associated ligand capable of synergizing with IL-7 signals to promote the proliferation and responsiveness of pathogen-specific T cells. We speculate that self-Ag/TCR-initiated signals may play a role in the IL-7 cultures.

Although further research is needed to identify such signals in IL-7 cultures, our data support a role for IL-7 in T cell-driven immunopathology in chronic and persistent infections, coinfections, or autoimmunity. Accordingly, IL-7 is expressed in inflamed tissues of patients with (rheumatic) autoimmune diseases, where it can be produced by several cell types [43] (including macrophages, dendritic cells, and fibroblasts) and favor pathogenic Th1- and Th17-associated cytokine secretion. Furthermore, dysregulated IL-7 expression or activation of CD127 were found in patients with autoimmune conditions [8–13], suggesting that IL-7 supports the function of pathogenic effector cells in autoimmunity. In agreement with this, blocking the IL-7R in experimental animal models ameliorated autoimmune disease manifestations [44]. Thus, together with available data, our results suggest that IL-7 might awaken auto-reactive T cells, or pathogen-specific effector T cells with cross-reactivity to self-Ag, hence contributing to autoimmunity. However, this might not equally apply to patients with immune dysfunctions due to sepsis or chronic HIV/hepatitis C virus infection. Indeed, IL-7 administration mainly in HIV-infected or immunosuppressed patients was generally well tolerated [22, 23, 25, 27, 28, 45], with a single report of a patient developing systemic lupus erythematosus after 3 doses of IL-7 [45]. We speculate that the risk of developing autoimmunity after IL-7 treatment may vary dependent on individual clinical history, genetic predisposition, and the administration regimen. Further studies are needed to define the long-term consequences of IL-7 administration.

It is interesting to note that among memory cells, polyfunctional T cells (double positive for IFNγ^+^ and IL-2^+^ or TNFα^+^) were mostly enriched for by IL-7. Such cells were detected in subjects with chronic viral (including CMV [32]) infections and previously referred to as intermediate polyfunctional memory cells [46]. It is possible that IL-7 favors differentiation of these cells in vitro (and possibly also in vivo), starting from IFNγ-producing cells. With respect to maintaining polyfunctional T cells, IL-7 appears superior to the cognate Ag by favoring (central) memory cell survival [15–17], rather than terminal differentiation, activation-induced cell death, and/or exhaustion [29]. Thus, IL-7 may be useful for the expansion of human polyclonal and polyfunctional pathogen-specific CD4 (and to a lower extent, CD3^+^CD4^−^ or CD8^+^; Supplementary Figure 8A–E) T cells that are hard to identify, even in the case of a relatively well studied pathogen (such as CMV [47, 48]). Accordingly, exposing cells to IL-7 enabled better enumeration of in vivo-primed CMV-/SEB-specific cells, although these trends were less apparent in CD8^+^ T cells, which did not undergo fast proliferation to the extent of CD4 T cells (Supplementary Figure 8F). This was also the case when T cells were Ag-restimulated in vitro before the IL-7 culture, opening the possibility that IL-7 (with or without Ag) might be superior to Ag alone in expanding T cells derived from in vivo-primed individuals. Our data also support the hypothesis that IL-7 sustains the preferential accumulation of polyfunctional T-cell subsets within the repertoire of certain individuals, including perhaps the inflated responses of CD4 T cells detected in elderly CMV^+^ individuals [32, 33]. Future studies are needed to address this possibility.

## CONCLUSIONS

We previously suggested that among other CD132-cytokines, IL-7 played nonredundant roles and outperformed IL-2 in driving Ag-experienced T-cell accumulation and mediating the expansion of less differentiated cells useful for gene therapy [26, 30]. We now provide evidence supporting the use of IL-7 to reveal and expand in vivo-primed pathogen-specific lymphocytes of clinical relevance, either as biomarkers of viral infection and disease activity [49] or as therapeutic tools. This may be relevant for the treatment of chronic infectious diseases and cancer, because adoptive immunotherapy with less differentiated T cells is preferable over the transfer of terminally differentiated effectors [50].

## Supplementary Data

Supplementary materials are available at *The Journal of Infectious Diseases* online. Consisting of data provided by the authors to benefit the reader, the posted materials are not copyedited and are the sole responsibility of the authors, so questions or comments should be addressed to the corresponding author.

Supplementary Figure 1

Supplementary Figure 2

Supplementary Figure 3

Supplementary Figure 4

Supplementary Figure 5

Supplementary Figure 6

Supplementary Figure 7

Supplementary Figure 8

## Notes

***Acknowledgments.*** We thank George Morrow and Dr. Helen Stewart (University of Sussex) for technical support and reading the manuscript, respectively. We also thank Antonio Lanzavecchia and members of the San Raffaele Institute for useful discussions and suggestions.

***Disclaimer.*** The funders had no role in study design, data collection and analysis, decision to publish or preparation of the manuscript.

***Financial support.*** S. C. is supported by a Brighton and Sussex Medical School Internal Fellowship (University of Sussex). This work was funded by grants from the following: “Associazione Italiana Ricerca sul Cancro (AIRC)”; the “Compagnia San Paolo IMI”; the Italian “Ministero dell’Istruzione, dell’Università e della Ricerca, Fondo per gli Investimenti della Ricerca di Base” (grant number RBNE017B4C_006; to A. M.); the University of Brighton PaBS Output Enhancement Fund to N. T.; and the University of Sussex Research Development Fund (grant number RDF 3-021; to S. C.).

***Potential conflicts of interest.*** S. C. and A. M. are inventors in a patent application for the use of common-ɣ chain cytokines for the visualization, isolation and genetic modification of memory T lymphocytes (US 2010.0035282A1). All authors have submitted the ICMJE Form for Disclosure of Potential Conflicts of Interest. Conflicts that the editors consider relevant to the content of the manuscript have been disclosed.

Presented in part: 4th European Congress of Immunology, 6–9 September 2015, Vienna, Austria.
