# Supplementary material for: Interleukin-7 Unveils Pathogen-Specific T Cells by Enhancing Antigen-Recall Responses
Source: J Infect Dis. 2018 Feb 28;217(12):1997–2007. doi: 10.1093/infdis/jiy096 (PMC5972594; doi:10.1093/infdis/jiy096)
Supplement: Supplementary Figure 1 [file jiy096_suppl_supplementary_figure_1.pdf]

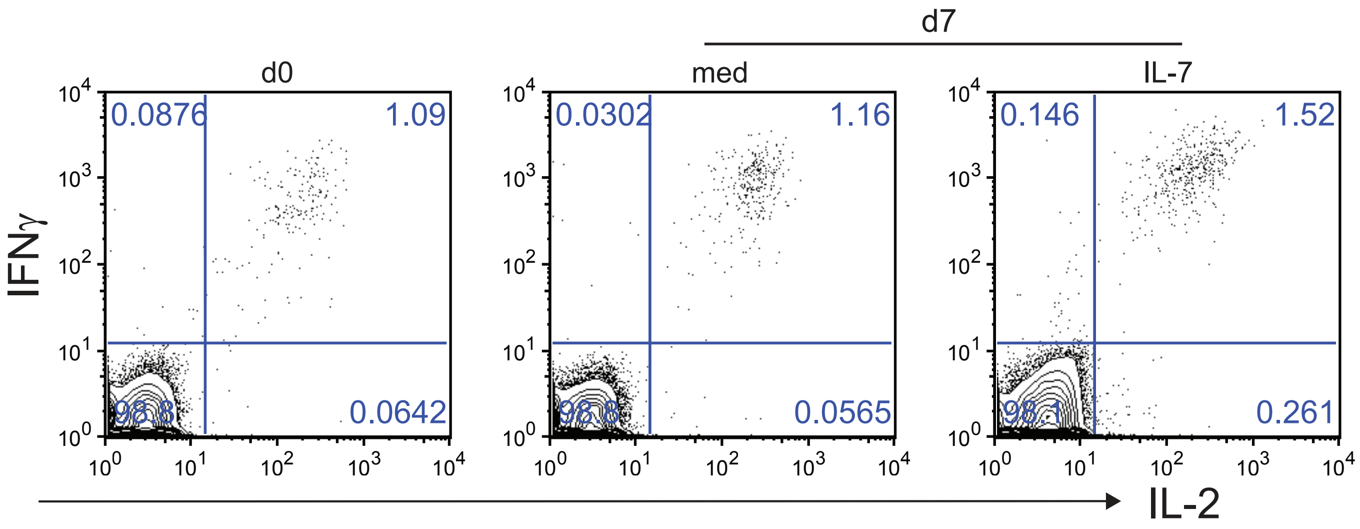

Supplementary Figure 1. IL-7 promotes the accumulation of MTB specific CD4 T cells over the levels found ex vivo. Thawed PBMCs of Pt. #1 (active TB) were analyzed at the time of thawing (d0) and after a 7-day culture in plain medium (d7, med), in IL-7 alone (d7, IL-7). Cells were stimulated MTP-pulsed autologous irradiated PBMCs and IL-2 and IFNγ release was determined by intracellular staining. Events are shown after gating on viable $CD4^+$ T cells.
