# Supplementary material for: Interleukin-7 Unveils Pathogen-Specific T Cells by Enhancing Antigen-Recall Responses
Source: J Infect Dis. 2018 Feb 28;217(12):1997–2007. doi: 10.1093/infdis/jiy096 (PMC5972594; doi:10.1093/infdis/jiy096)
Supplement: Supplementary Figure 2 [file jiy096_suppl_supplementary_figure_2.pdf]

A

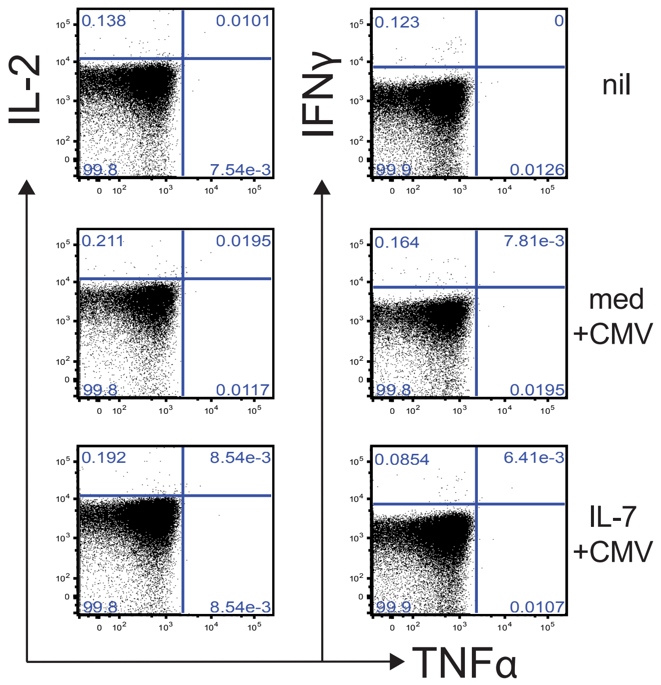

B

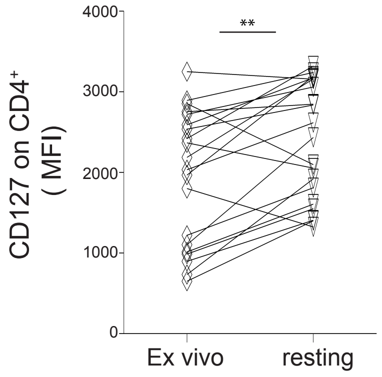

Supplementary Figure 2. IL-7 does not expand CMV-specific CD4 T cells from CMV$^-$ donors and a resting step in control medium drives the upregulation of CD127 expression in CD4$^+$ T cells. A. Freshly-derived PBMCs from CMV$^-$ donors were analyzed for IFNγ, IL-2 and TNFα release after CMV-lysate ICS assay after a 7-day culture in the absence (d7, med+CMV) or in the presence of human recombinant IL-7 (d7, IL-7+CMV). Typically very low or null levels of cytokines were detected in non-re-stimulated controls (nil, upper panels). Data are representative of 9 independent biological repeats. **B.** Freshly isolated PBMCs were rested for 2 days in control medium. At d2 (resting), the levels of expression of CD127 (Mean Fluorescence Intensity, MFI) in CD4$^+$ T cells were measured in comparison to the levels expressed ex vivo, at the start of the culture (d0). CD127 expression is significantly upregulated in CD4$^+$ T cells rested in control medium (resting) across many individuals (n=20, Wilcoxon matched-pairs signed rank test, p=0.003).
