# Supplementary material for: Interleukin-7 Unveils Pathogen-Specific T Cells by Enhancing Antigen-Recall Responses
Source: J Infect Dis. 2018 Feb 28;217(12):1997–2007. doi: 10.1093/infdis/jiy096 (PMC5972594; doi:10.1093/infdis/jiy096)
Supplement: Supplementary Figure 3 [file jiy096_suppl_supplementary_figure_3.pdf]

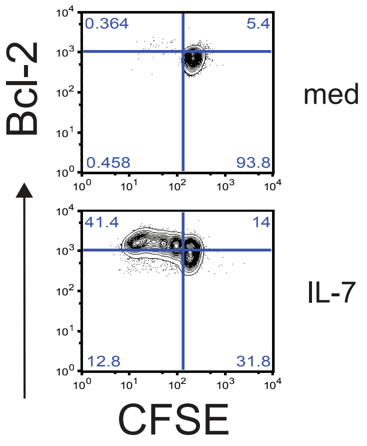

Supplementary Figure 3. Cells cultured in IL-7 upregulate Bcl-2 while proliferating in vitro. CFSE-labeled PBMCs derived from healthy donors were cultured ($5x10^6$ cells/ml) for 7 days with (IL-7) or without IL-7 (med). At d7, proliferation was assessed alongside the expression of the pro-survival mediator (Bcl-2) in flow cytometry. The small frequency of cells autoproliferating in the absence of IL-7 tended to show higher Bcl-2 levels than not-dividing CD4 T cells. Addition of IL-7 supported the proliferation of CD4 T cells alongside the upregulation of the anti-apoptotic factor, Blc-2.
