# Supplementary material for: Interleukin-7 Unveils Pathogen-Specific T Cells by Enhancing Antigen-Recall Responses
Source: J Infect Dis. 2018 Feb 28;217(12):1997–2007. doi: 10.1093/infdis/jiy096 (PMC5972594; doi:10.1093/infdis/jiy096)
Supplement: Supplementary Figure 4 [file jiy096_suppl_supplementary_figure_4.pdf]

A

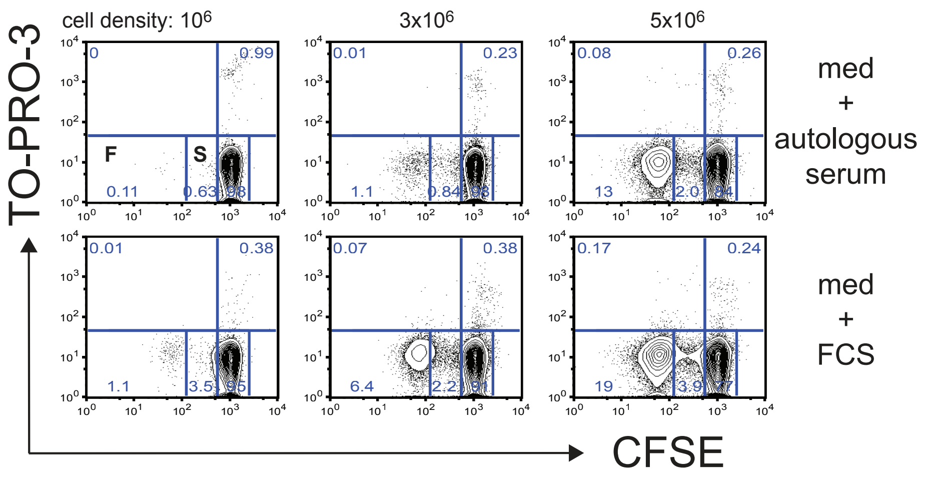

B

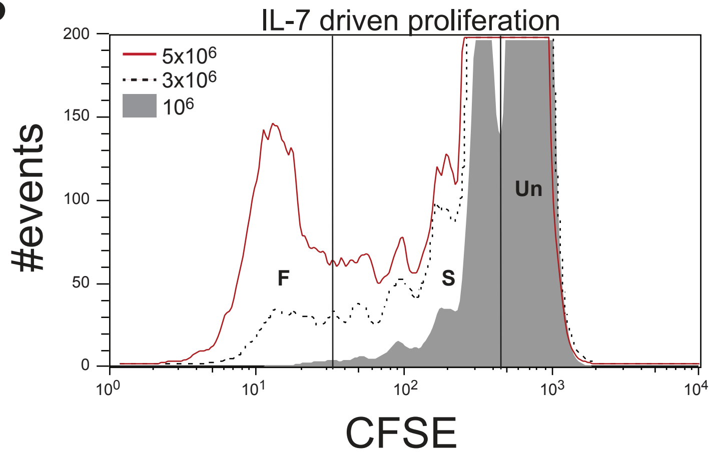

Supplementary Figure 4. Cell-density dependent spontaneous proliferation of a fraction of human peripheral blood CD4$^+$ T cells, in cultures derived in the presence of autologous serum. Healthy donor PBMCs were stained with the CSFE vital dye in the presence of autologous serum (A) or FCS (A-B) during all the necessary steps (i.e. the quenching of the labelling reaction as well as the final washes, as specified in Material and Methods). **A.** CFSE-labelled cells were cultured for 7 days in control medium (med) at the indicated cell densities (1-5x10^6 cells/ml), in the presence of autologous serum (top row) or FBS (bottom row). At d7, proliferation was assessed alongside cell viability by measuring the CFSE content in parallel to the amount of TO-PRO-3 (an intercalant agent of DNA entering necrotic and apoptotic cells) dye incorporated in CD4$^+$ T cells. Representative dot plots of viable CD4$^+$ T cells are depicted. The frequency of CD4$^+$ TO-PRO-3$^-$ CFSEdim T cells is shown. **B.** CFSE-labelled cells were cultured for 7 days in IL-7 at the indicated cell densities (1-5x10^6 cells/ml). At d7, proliferation was assessed as shown in the histogram overlay gated on similar CD4$^+$ T cell numbers. The scale of the plot has been optimized to allow the comparison of proliferating cells in the different conditions. Fast- (F), Slow- (S), and non-dividing cells.
