# Supplementary material for: Interleukin-7 Unveils Pathogen-Specific T Cells by Enhancing Antigen-Recall Responses
Source: J Infect Dis. 2018 Feb 28;217(12):1997–2007. doi: 10.1093/infdis/jiy096 (PMC5972594; doi:10.1093/infdis/jiy096)
Supplement: Supplementary Figure 5 [file jiy096_suppl_supplementary_figure_5.pdf]

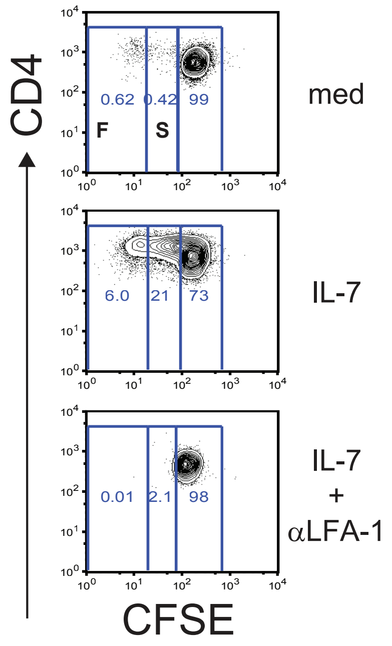

Supplementary Figure 5. Blocking the LFA-1/ICAM-1 interaction abrogates the cell density-dependent, IL-7-driven proliferation. CFSE-labeled PBMCs derived from healthy donors were cultured (4x10^6 cells/ml) for 7 days with (IL-7) or without IL-7 (nil) and in the presence of IL-7 plus anti-LFA-1 blocking antibody, as in Figure 5B. Proliferation in high density cultures was determined by flow cytometry after staining with anti-CD4 mAb. Dot plots depict the relative CFSE content within the same number of viable $CD4^+$ T cells. Fast- (F), Slow- (S), and Non- (N) dividing cells.
