# Supplementary material for: Interleukin-7 Unveils Pathogen-Specific T Cells by Enhancing Antigen-Recall Responses
Source: J Infect Dis. 2018 Feb 28;217(12):1997–2007. doi: 10.1093/infdis/jiy096 (PMC5972594; doi:10.1093/infdis/jiy096)
Supplement: Supplementary Figure 6 [file jiy096_suppl_supplementary_figure_6.pdf]

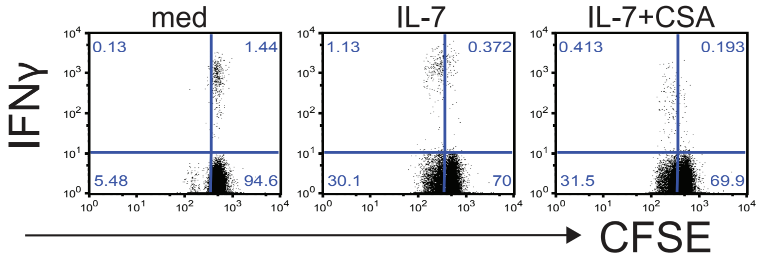

Supplementary Figure 6. Sensitization by IL-7 promotes MTB-specific CD4$^+$ T cells proliferation in CSA-sensitive manner. CFSE-labelled PBMCs derived from TB Pt.#1 (as shown in Fig.1B) were analyzed after a 7-day culture in IL-7 (IL-7) and IL-7 plus CSA (IL-7+CSA) compared to control medium (med). At d7, cells were stimulated with MTP-pulsed autologous irradiated PBMCs and IFNγ release was determined by ICS. Events are shown after gating on viable CD4$^+$ T cells.
