# Supplementary material for: Interleukin-7 Unveils Pathogen-Specific T Cells by Enhancing Antigen-Recall Responses
Source: J Infect Dis. 2018 Feb 28;217(12):1997–2007. doi: 10.1093/infdis/jiy096 (PMC5972594; doi:10.1093/infdis/jiy096)
Supplement: Supplementary Figure 7 [file jiy096_suppl_supplementary_figure_7.pdf]

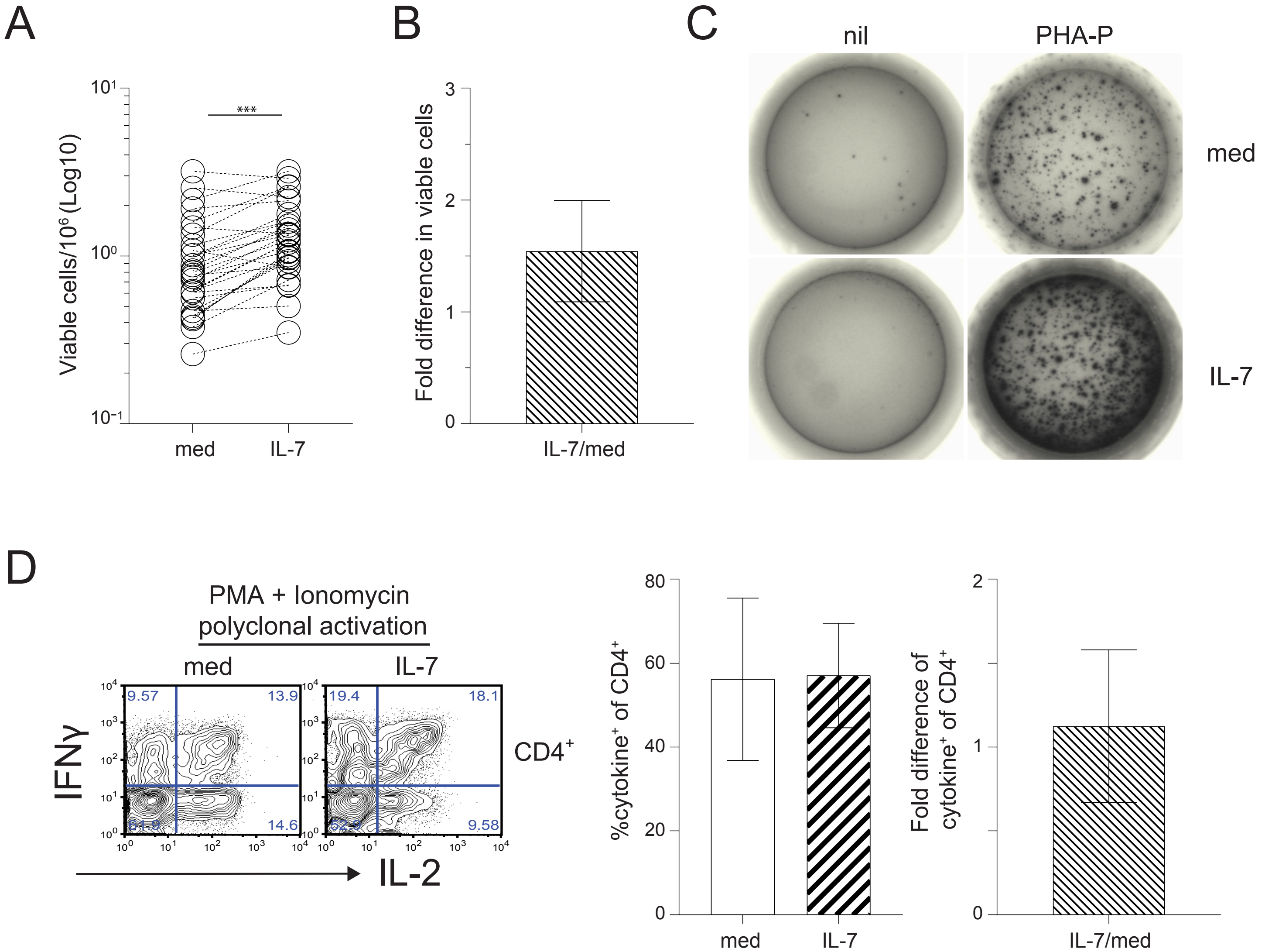

Supplementary Figure 7. IL-7 enhances total cell recovery and non-specific IFNγ secretion, but does not increase the total cytokine secretion by CD4 T cells. (A-D) At d0, equal number of viable PBMCs were seeded in culture with IL-7 (IL-7) and control media (med). (A-B) After a 7-day culture, alive cells were counted as 0.1% Trypan Blue negative cells. **A.** The plot shows total viable cell recovery in complete media (med) compared to IL-7 (IL-7) in many independent biological replicates (n=30). IL-7 significantly enhanced total cell recovery ($p<0.0001$, Wilcoxon matched-pair test). **B.** For each independent pair (n=30), the fold-increase in viable cells was calculated as a ratio between the cell numbers detected in the culture in IL-7 compared to that in complete media (IL7/med). The graph shows that, on average, total viable cells increased by ~1.5 fold, upon exposure to IL-7. **C.** PBMCs derived from a representative donor were analyzed for the release of the effector cytokine, IFNγ by ELISPOT after a 7-day culture in the absence (med, upper wells) or in the presence of human recombinant IL-7 (IL-7, lower wells). IFNγ release was measured in unstimulated negative control wells (nil, wells on the left) compared to the ELISPOT positive control, PHA-P stimulation (wells on the right). In agreement with A-B, the total number of spots tended to increase after the IL-7 culture. **D.** In order to measure whether polyclonal CD4$^+$ T cells generally benefit from the IL-7 culture, PBMCs cultured for 7 days in complete media (med) or with IL-7 (IL-7) were stimulated with PMA and Ionomycin (polyclonal stimulation: 50 ng/ml and 1 μg/ml, respectively) in ICS assay (refer to Materials and Methods for details). Then, polyclonal IFNγ/IL2 release was analyzed in gated CD4$^+$ T cells (left contour plots). When average percentages of total cytokine$^+$ (IFNγ$^+$ and/or IL2$^+$) CD4$^+$ T cells were evaluated in cultures derived from 5 independent donors (middle bar graph), no significant difference was observed in IL-7 (black striped bar) compared to control medium (white bar) cultures (Wilcoxon matched-pairs signed rank test). The right graph shows the average ratio of percentage of cytokine$^+$ CD4$^+$ T cells between the IL-7 and control media culture (IL7/med).
