# Supplementary material for: Interleukin-7 Unveils Pathogen-Specific T Cells by Enhancing Antigen-Recall Responses
Source: J Infect Dis. 2018 Feb 28;217(12):1997–2007. doi: 10.1093/infdis/jiy096 (PMC5972594; doi:10.1093/infdis/jiy096)
Supplement: Supplementary Figure 8 [file jiy096_suppl_supplementary_figure_8.pdf]

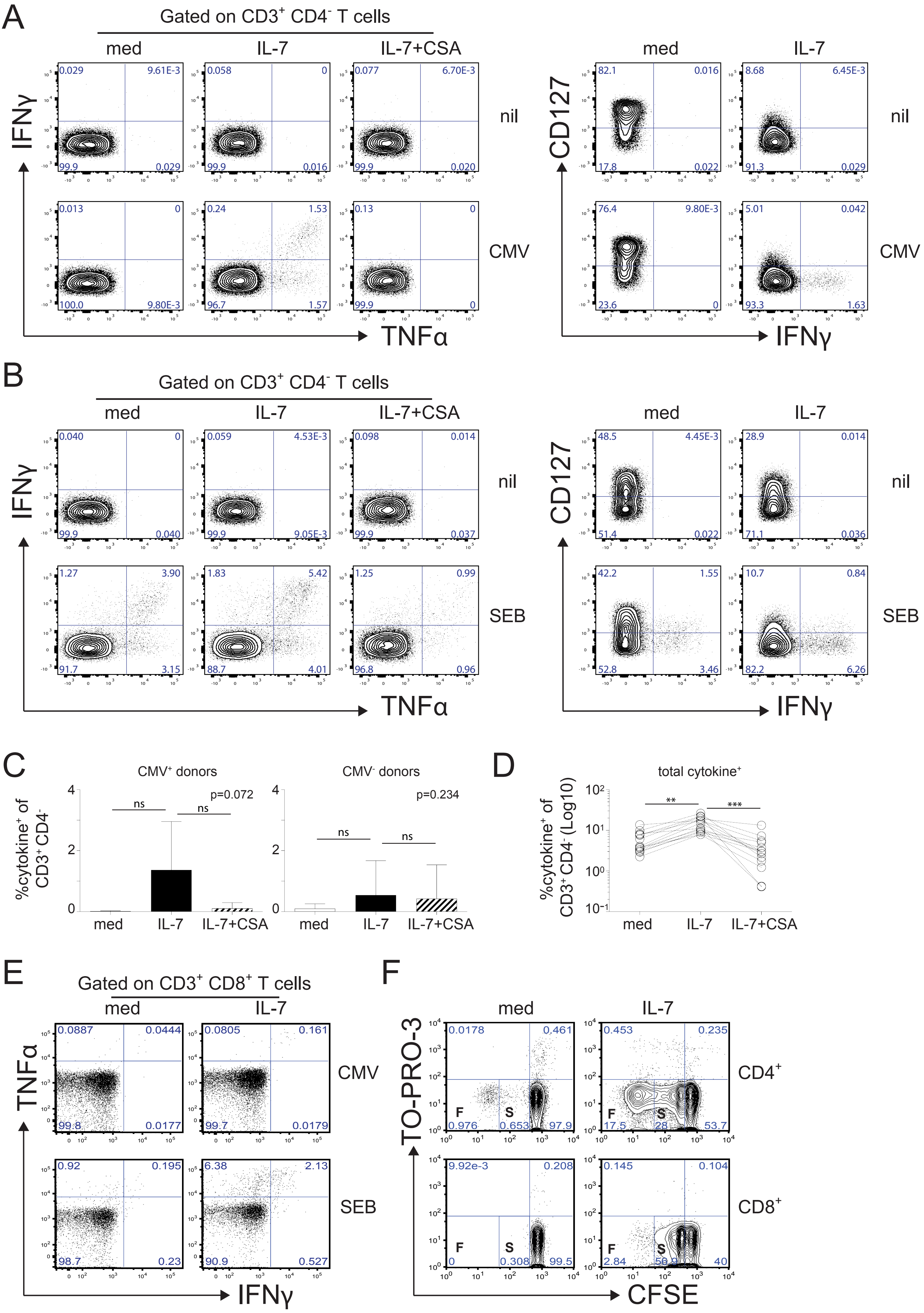
A
Gated on CD3+ CD4- T cells
med
IL-7
IL-7+CSA
nil
CMV
IFNγ
TNFα
CD127
IFNγ
B
Gated on CD3+ CD4- T cells
med
IL-7
IL-7+CSA
nil
SEB
IFNγ
TNFα
CD127
IFNγ
C
CMV+ donors
CMV- donors
%cytokine+ of CD3+ CD4-
p=0.072
p=0.234
ns
med
IL-7
IL-7+CSA
D
total cytokine+
%cytokine+ of CD3+ CD4- (Log10)
**
***
med
IL-7
IL-7+CSA
E
Gated on CD3+ CD8+ T cells
med
IL-7
CMV
SEB
TNFα
IFNγ
F
med
IL-7
CD4+
CD8+
TO-PRO-3
CFSE

Supplementary Figure 8. Sensitization by IL-7 also promotes CMV and SEB-specific CD8$^+$ T cells accumulation in CSA-sensitive manner. (A-D) Freshly isolated PBMCs were rested for 5 days in plain medium. At d5, cells were harvested, washed and counted. Then, equal number of cells were incubated for an additional week in IL-7 (IL-7) or control medium (med) (as shown in Fig. 3B and 4A, respectively for CMV and SEB responses), in the presence or absence of CSA. At d12, cells were stimulated with CMV or SEB during an ICS assay to test Ag-specific TNFα/IFN γ release and surface marker (CD127) expression, compared to unstimulated controls (nil). (A-B) Contour plots (left) show that the frequency of TNFα$^+$/IFNγ$^+$ CMV- (**A**) and SEB-specific (**B**) CD3$^+$ CD4$^-$ T cells increased upon exposure to IL-7, in a representative donor. The IL-7-driven increase of cytokine$^+$ CD3$^+$ CD4$^-$ T cells is reversed in the presence of CSA (IL7+CSA). Right contour plots (gated on CD3$^+$ CD4$^-$) show the expression of CD127 in CMV- (A) and SEB-specific (B), IFNγ$^+$ CD3$^+$ CD4$^-$ cells compared to IFNγ$^-$, non-responder cells from the same cultures. **C.** Graphs show percentage of total cytokine$^+$ CD3$^+$ CD4$^-$ T cells in cultures derived from independent CMV$^+$ (n=7, left) and CMV$^-$ (n=7, right) donors (after subtraction of individual background levels of cytokine$^+$ CD3$^+$ CD4$^-$ T cells). Although a trend towards increased cytokine secretion is apparent in CMV$^+$ donors, there is no significant difference in IL-7 compared to control medium (med) cultures derived from CMV$^+$ (p=0.072) and CMV$^-$ (p=0.234) donors (Friedman test, non-parametric ANOVA). **D.** After subtraction of individual background levels of cytokine$^+$ CD3$^+$ CD4$^-$ T cells detected in unstimulated controls (nil), the percentage of SEB-specific cytokine$^+$ CD3$^+$ CD4$^-$ T cells was evaluated in 14 independent biological replicates. The graph shows that the frequency (Log10) of SEB-specific cytokine$^+$ CD3$^+$ CD4$^-$ T cells significantly increased upon IL-7 treatment, and this was significantly inhibited by CSA (Friedman test, non-parametric ANOVA and Dunn's multiple comparisons test). **E.** Equal number of freshly isolated PBMCs were incubated for a week in IL-7 (IL-7) or control medium (med) (as shown in Fig. 1, 2 and 3A). At d7, cells were stimulated with CMV or SEB during an ICS assay to test Ag-specific cytokine release in CD8$^+$ T cells, directly. Dot plots (gated on CD3$^+$ CD8$^+$ T cells) show a trend to increased frequency of TNF α$^+$/IFNγ$^+$ in CMV- (top) and SEB-specific (bottom) CD3$^+$ CD8$^+$ T cells after IL-7 exposure. **F.** CFSE-labeled PBMCs from healthy donors were cultured for 7 days in the absence (med) or in the presence of IL-7 (IL-7). Proliferation of viable T cells in high-density cultures ($5x10^6$ cells/ml) was determined by flow cytometry, after staining with anti-CD8 and anti-CD4 mAb and TO-PRO-3 (an intercalant agent of DNA entering necrotic and apoptotic cells). Contour plots show the relative CFSE content in gated CD4$^+$ (top) and CD8$^+$ (bottom) cells: fast- (F), slow- (S), and non-dividing cells were determined, as indicated. CD8$^+$ T cells did not undergo fast-proliferation to the same extent of their CD4$^+$ counterparts, in both IL-7 and control medium cultures.
